# A systematic literature review on spam content detection and classification

**DOI:** 10.7717/peerj-cs.830

**Published:** 2022-01-20

**Authors:** Sanaa Kaddoura, Ganesh Chandrasekaran, Daniela Elena Popescu, Jude Hemanth Duraisamy

**Affiliations:** 1 Zayed University, Abu Dhabi, United Arab Emirates; 2 Electronics and Communication Engineering, Sri Eshwar College of Engineering, Coimbatore, Tamil Nadu, India; 3 Faculty of Electrical Engineering and Information Technology, University of Oradea, Oradea, Romania; 4 Electronics and Communication Engineering, Karunya Institute of Technology and Sciences, Coimbatore, Tamil Nadu, India

**Keywords:** Spam Content, Machine learning, Deep learning, Natural language processing, Social media analysis, Classification, Text mining, Data mining

## Abstract

The presence of spam content in social media is tremendously increasing, and therefore the detection of spam has become vital. The spam contents increase as people extensively use social media, *i.e*., Facebook, Twitter, YouTube, and E-mail. The time spent by people using social media is overgrowing, especially in the time of the pandemic. Users get a lot of text messages through social media, and they cannot recognize the spam content in these messages. Spam messages contain malicious links, apps, fake accounts, fake news, reviews, rumors, etc. To improve social media security, the detection and control of spam text are essential. This paper presents a detailed survey on the latest developments in spam text detection and classification in social media. The various techniques involved in spam detection and classification involving Machine Learning, Deep Learning, and text-based approaches are discussed in this paper. We also present the challenges encountered in the identification of spam with its control mechanisms and datasets used in existing works involving spam detection.

## Introduction

The word spam generally means some unwanted text sent or received through social media sites such as Facebook, Twitter, YouTube, e-mail, etc. It is generated by spammers to divert the attention of the users of social media for the purpose of marketing and spreading some malware etc. The e-mail spam messages are sent in bulk to various users, with the intention of tricking them into clicking on fake advertisements and spreading malware on their devices. The spam messages provide a good source of income for the spammers (Bauer, 2018) and, hence, they continue to spread them rapidly. To combat spam in e-mail, a lot of techniques have been involved, but the spam content continues to increase (Statista, 2017). These spam messages cause financial loss to business e-mail consumers and also to the general users of e-mail (Okunade, 2017).

Spam is common on social media sites like YouTube, and it mainly consists of comments and links to pornographic websites, as well as irrelevant videos. These comments are sometimes created automatically by bots. Although the definition of spam on online video game sharing services is debatable, instances of message flooding, requests to join a specific group, violations of copyrights, and so on are occasionally referred to as spam. Spam in blogs, often known as splog, refers to comments that have nothing to do with the topic of discussion. Frequently, these comments are accompanied by links to commercial websites. Some splogs are devoid of unique content and contain stuff plagiarized from other websites (Rouse, 2015).

Spam is also included in written reviews of products that are available on social networking sites. According to Liu & Pang (2018), about 30–35% of online reviews are deemed spam. These spam reviews are intended to influence people’s purchasing decisions and to affect product ratings (Saini, Saumya & Singh, 2017; Ho-Dac, Carson & Moore, 2013). As a result, detecting bogus reviews appears to be a major worry, and online review systems may become utterly useless unless this vital issue is addressed (Jin et al., 2011; Govtnaukries, http://www.govtnaukries.com/you-wont-ever-use-head-and-shoulder-shampoo-after-watching-this-video-facebook-spam/). Fake/spam profiles abound on social networking platforms like Facebook and Twitter, and users are bombarded with SMS messages from these identities. To analyze the spam content many researchers Song, Lee & Kim (2011) have employed the attributes from Facebook including community, URL, videos and Images. By identifying and filtering the spam and non-spam accounts Stringhini, Kruegel & Vigna (2010) could identify and characterize the spam using statistical techniques. Mateen et al. (2017) have used honey-profiles to record the activity of the spammers and applied this technique to social media content for spam detection using a novel tool. The graph models were also popular to detect spam based on the different features of the map and they could find the relationships that exist among the social media users (Benevenuto et al., 2010). In recent times, the machine learning algorithms are getting popular and they are used in spam detection (Rathore, Loia & Park, 2018; Liu et al., 2016; Zheng et al., 2016; Serrano-Guerrero et al., 2015).

The steps in detecting spam on social media are often as follows. Obtaining the spam text collection (dataset) is the initial step. Because these datasets frequently have unstructured text and may contain noisy data, preprocessing is almost always necessary. The following step is to select a feature extraction method, such as Word2Vec, n-grams, TF-IDF, and so on. Finally, a variety of spam detection technologies, such as machine learning, deep learning, and Lexicon-based algorithms, are utilized to decide whether texts are spam.

The rationale of our work is to bring out a detailed survey of several spam detection and categorization algorithms. We are aware that many previous surveys on spam detection may not have acquired the information that we obtained from various popular academic data sources. Some previous efforts on spam identification from social media have constrained themselves to only a few limited academic sources. Some earlier studies failed to highlight the benefits and drawbacks of various spam detection and classification systems. The novelty of our work is that we used data from a variety of reputable academic sources to achieve our goal of identifying spam content on social media. We have also highlighted certain significant strategies, along with their benefits and drawbacks when applied to various spam datasets. We also covered deep learning and other crucial Artificial Intelligence (AI)-based spam detection approaches that have previously only been found in restricted investigations.

This extensive survey will assist academics who are interested in spotting social media spam using AI techniques, as well as addressing the issues associated with it. Using the proposed survey, researchers will be able to select optimal detection and control mechanisms for spam eradication. Our work will let academics compare the many existing spam detection works in terms of their merits, limits, approaches, and datasets employed. This study will also assist researchers in addressing current research possibilities, concerns, and challenges connected to spam text feature extraction and classification, as well as specifics on various data sets used by other researchers for spam text detection.

We compare the accuracy of existing spam text detection systems in order to determine which ones are the most effective. “Survey Methodology” describes the survey methodology used to conduct our comprehensive review. “Steps for Detecting Spam in Social Media Text” uses a block diagram to explain the multiple steps involved in spam detection. “Collection of Social Media Textual Data (Dataset Collection)” provides a summary of the datasets available for social media spam text. The following section, “Pre-processing of Textual Data”, goes over the various spam text pre-processing procedures. “Feature-Extraction Techniques” and “Spam Text Classification Techniques” investigate several feature extraction methodologies and spam categorization algorithms. Deep learning techniques for spam classification are discussed in “Deep Learning (DL) Approaches for Spam Classification”. “Challenges in Spam Detection/classification from Social Media Content” discusses the difficulties encountered in spam detection, and “Open Issues and Future Directions” concludes with a list of references.

## Survey methodology

The goal of this survey is to undertake a thorough literature evaluation on approaches for detecting and classifying spam content in social media. There are several sources of textual data on social media platforms such as Facebook, Twitter, E-mail, and YouTube. A variety of ways have been used to detect and regulate spam text. Our efforts are primarily motivated by a desire to learn more about different spam text detection and categorization algorithms. This section discusses the survey methodology that we used to conduct our detailed spam detection review.

### Selection of keywords and data sources

Based on our research objective, the initial search keywords were carefully chosen. Following an initial search, new words discovered in several related articles were used to generate several keywords. These keywords were later trimmed to fit the research’s objectives. We chose certain search keywords based on the goal of our survey work, and after performing an initial search on those words, several keywords were derived from selected articles. The number of keywords is then reduced in order to meet our research goal.

### Database selection

We extracted research papers from a few academic digital sources to conduct the literature review. Expert advice was sought regarding source selection, and databases such as Web of Science (WoS), Scopus, Springer, IEEE Xplore, and ACM digital library were used to collect research papers for our study. We used search query terms such as “social media spam,” “twitter spam,” “review spam,” and “spam text,” among others. The academic data sources with their links that are used in our work is listed in the Table 1 below.

**Table 1:** Description about academic databases and their links.

Academic Data sources	Search string	Links
WoS	Social spam	https://apps.webofknowledge.com/
Scopus	Spam AND Twitter	https://www.scopus.com/
Springer	Spam AND Artificial Intelligence	https://link.springer.com/
IEEE Xplore	Social spam AND Artificial Intelligence	https://ieeexplore.ieee.org/
ACM Digital Library	Online spam AND Review Spam	http://dl.acm.org/
Science Direct	Social media AND Spam	http://www.sciencedirect.com/

**Figure 1:**
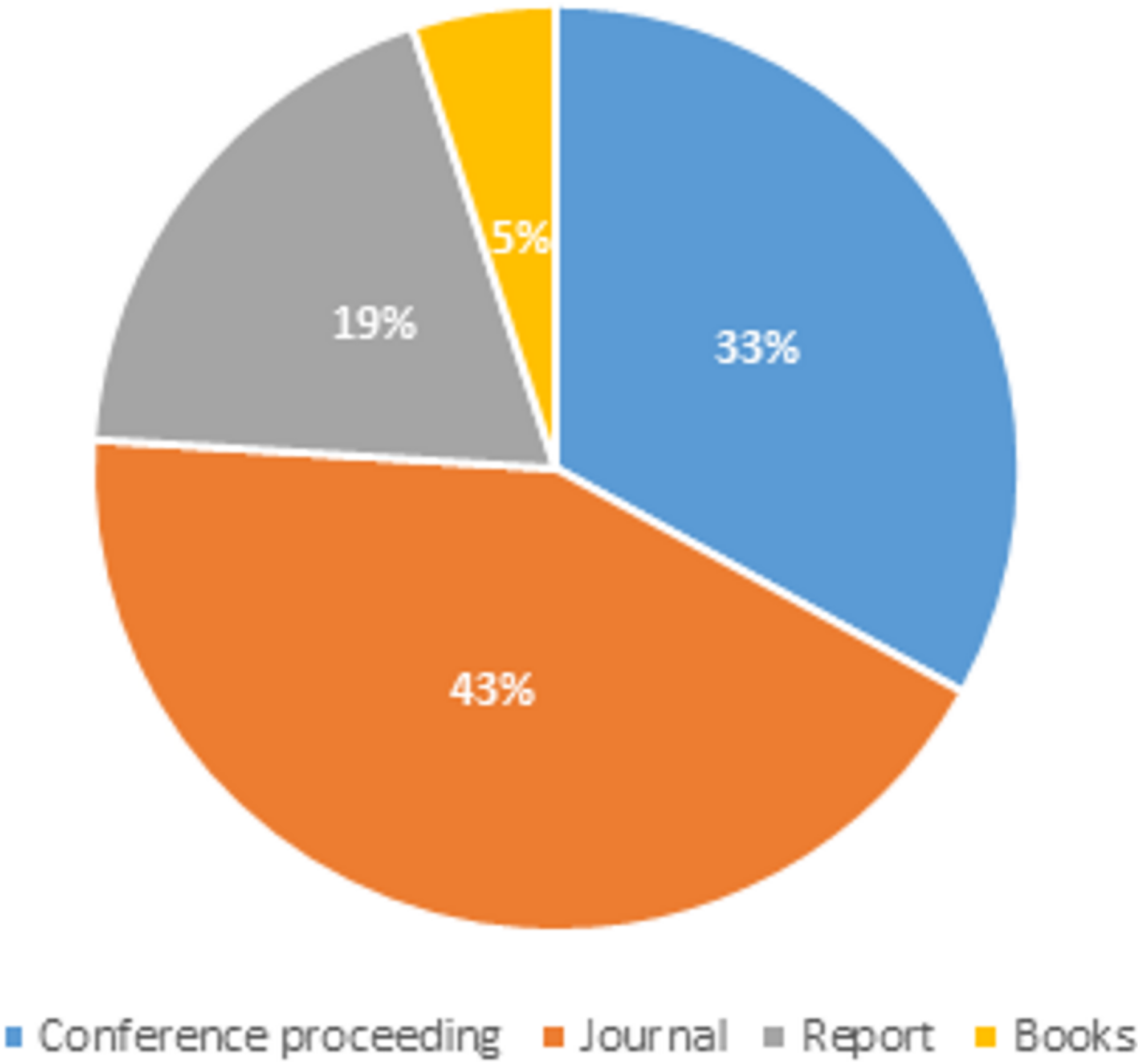
Articles distribution based on publication type.

In this review, the title of each paper was scanned and identified for possible relevance to this review. Any paper that does not refer to social media spam was eliminated from further investigation. The abstract and keywords of the publications were scanned for a deeper review and a better understanding of the papers. The Fig. 1 below displays the distribution of articles depending on publishing types such as journals, conference proceedings, books, and other reference materials that were referred for our extensive spam detection survey.

We may conclude from the article distribution pie-chart that for our work, the majority of the articles referred to were from journals and conference proceedings, and that some technical reports were also used to obtain material for our systematic literature review.

## Steps for detecting spam in social media text

**Figure 2:**
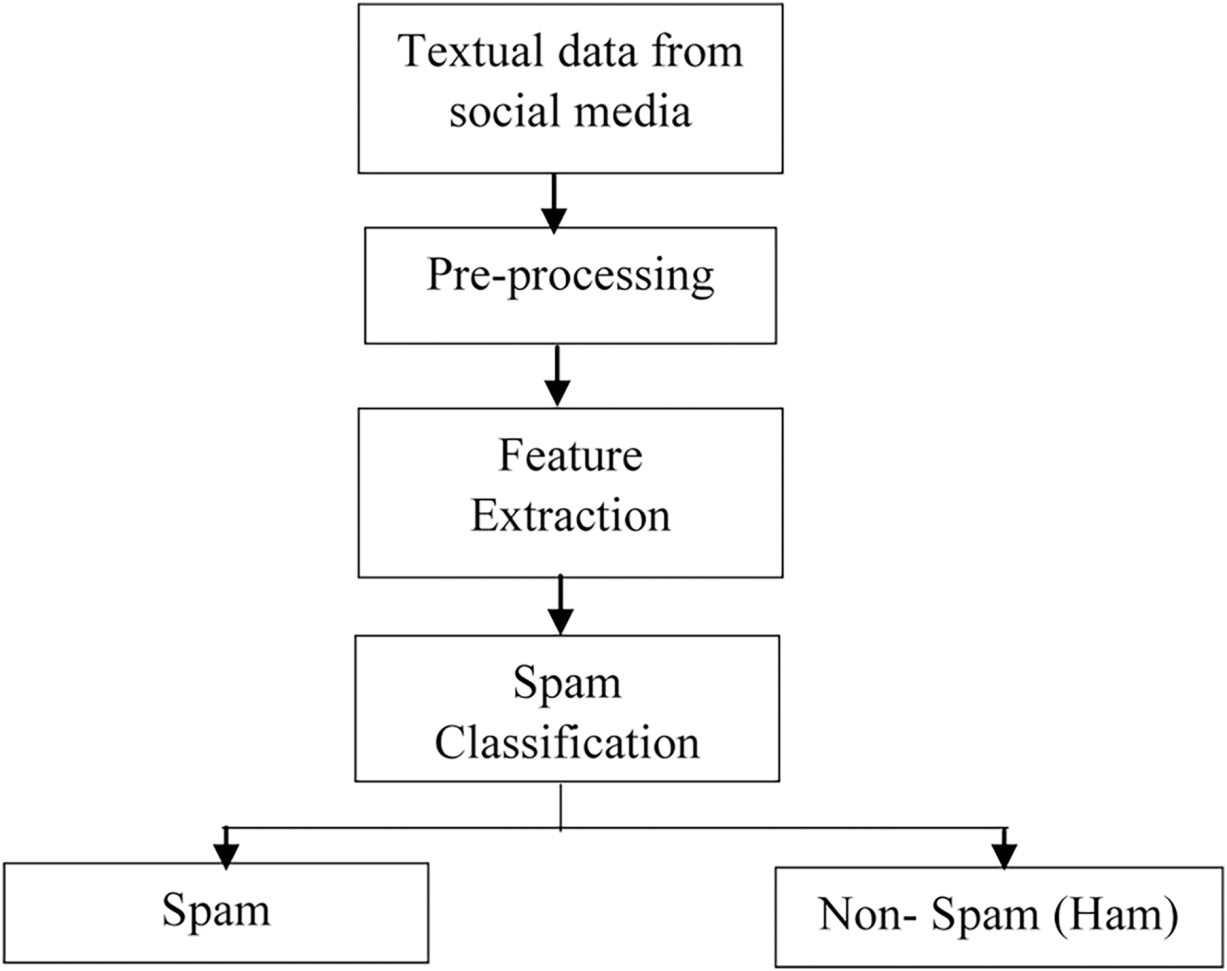
Steps in spam detection.

The task of spam detection and classification requires several processes, as depicted in Fig. 2. Data is collected in the first stage from social networking sites such as Twitter, Facebook, e-mail, and online review sites. Following data collecting, the pre-processing activity begins, which employs several Natural Language Processing (NLP) approaches to remove the unwanted/redundant data. The third phase entails extracting features from the text data using approaches such as Term Frequency-Inverse Document Frequency (TF-IDF), N-grams, and Word embedding. These feature extraction/encoding approaches convert words/text into a numerical vector that can be used for classification.

The last step is the spam detection phase, which employs several Machine Learning (ML) and Deep Learning techniques to classify the text into categories like spam and non-spam (ham).

## Collection of social media textual data (dataset collection)

The first phase in spam identification is the collecting of textual data, comprising spam and non-spam (ham) material, from social media sites such as Twitter, Facebook, online reviews, hotel evaluations, and e-mails. They are extracted with the help of an appropriate API, such as the Facebook API or the Twitter API, which are both free and allow users to search and collect data from several accounts. They also enable the capture of data using a “hashtag” or “keyword,” as well as the collecting of data posted over time. Based on the text content, we can identify data as spam or ham, and official social networking sites may flag some accounts or postings as spam. The following Table 2 presents some of the datasets regarding E-mail spam and Twitter spams. It also displays a description of the dataset as well as some of the reference studies performed on those datasets.

**Table 2:** E-mail spam datasets with their description.

S. No	Dataset name	Description	Reference	Web link
1	Spam Assassin	1,897 spam and 4,150 ham messages	(Méndez et al., 2006)	https://spamassassin.apache.org/old/publiccorpus/
2	Princeton Spam Image Benchmark	1,071 spam images	(Biggio et al., 2011)	https://www.cs.princeton.edu/cass/spam/
3	Dredze Image Spam Dataset	3,927 spam and 2,006 spam images	(Almeida & Yamakami, 2012)	https://www.cs.jhu.edu/~mdredze/datasets/image_spam/
4	ZH1–Chinese email spam dataset	1,205 spam and 428 ham text emails	(Zhang, Zhu & Yao, 2004)	https://archive.ics.uci.edu/ml/datasets/spambase
5	Enron-Spam	13,496 spam and 16,545 non spam email text	(Koprinska et al., 2007)	http://www2.aueb.gr/users/ion/data/enron-spam/

Twitter, a prominent microblogging network, has attracted people from all around the world looking to express themselves through multimedia content. Spammers transmit uninvited information, including malware URLs and popular hashtags. Twitter suspends accounts that send a high volume of friend requests to people they don’t know, as well as accounts with a high number of followers but few followers. Table 3 below includes descriptions and references for some of the Twitter spam datasets.

**Table 3:** Twitter spam datasets with their description.

S. No	Dataset name	Description	Reference	Web link
1	Bzzfeednews dataset	11,000 labeled users, 1,000 spammers and 10,000 non-spammer users	(Mohale & Leung, 2018)	https://data.world/buzzfeednews
2	**Dataset1:** Buzzfeed Election Dataset **Dataset2:** Political news Dataset	Fake election news dataset with 36 real and 35 fake news stories 75 fake news stories	(Horne & Adalı, 2017)	https://data.world/buzzfeednews https://data.world/datasets/politics
3	Twitter ground labeled ground truth dataset	6.5 million spam and 6 million non-spam tweets	(Chen et al., 2015)	http://nsclab.org/nsclab/resources/
4	Twitter social honeypot dataset	22,223 spammers and 19,276 non-spammer users	(Lee, Caverlee & Webb, 2010)	http://infolab.tamu.edu/data/
5	Stanford Twitter sentiment 140 dataset	1.6 million tweets for spam detection with a total tweet id of 4435.	(Mazikua et al., 2020)	http://help.sentiment140.com/for-students

Sites such as TripAdvisor, Amazon, and Yelp, among others, have online reviews of a product, hotel, or movie. These reviews include input from previous customers who have purchased a product or stayed at a hotel. Spammers blend spam content with these reviews to convey a negative impression about a product or service, causing the firm financial harm. Table 4 below covers a few datasets linked to online reviews, as well as several reference studies on detecting spam in reviews.

**Table 4:** Spam review datasets with their description.

S. No	Dataset name	Description	Reference	Web link
1	Single Domain hotel review	1,600 hotel reviews (800 spam and ham) from TripAdvisor website belonging to 20 popular hotels in Chicago	(Ott, Cardie & Hancock, 2013)	https://github.com/Diego999/HotelRec
2	Multi-Domain review dataset	Hotels, Restaurant and Doctors reviews dataset (2,840 reviews)	(Li et al., 2014)	https://www.cs.jhu.edu/~mdredze/datasets/sentiment/
3	Yelp Review Dataset	85 hotels and 130 restaurant reviews in and around Chicago	(Mukherjee et al., 2013)	http://odds.cs.stonybrook.edu/yelpzip-dataset/
4	Store Review Dataset	4,08,470 reviews on 14,651 stores obtained from www.resellerratings.com	(Wang et al., 2011)	https://www.kaggle.com/mmmarchetti/play-store-sentiment-analysis-of-user-reviews/data
5	Amazon e-commerce Dataset	40,000 samples for training and 10,000 samples for testing were collected on various categories like Beauty, Fashion and Automotive etc.	(Salminen et al., 2022)	https://data.world/datasets/amazon
6	Hotel reviews dataset	42 fake and 40 hotel reviews	(Yoo & Gretzel, 2009)	https://www.cs.cmu.edu/~jiweil/html/hotel-review.html
7	Trustpilot company review dataset.	9,000 fake and real reviews from online company Trustpilot	(Sandulescu & Ester, 2015)	https://business.trustpilot.com/features/analyze-reviews

Table 5 below contains some of the most prevalent spam words seen in e-mail, Twitter, and Facebook posts. If your e-mail contains any of these words, it’s quite likely that it'll end up in the spam bin.

**Table 5:** Most often used spam terms in e-mail, Facebook, and Twitter.

S. No	Social network	Words
1	E-mail	Full refund, Get it Now, Order now, Order status, Make money, Earn extra cash, 100% free, Apply now, Click here, Sign up free, Winner, Lose weight, Lifetime, Gift certificate.
2	Twitter	Amazing, Hear, Watch, Hunt, Win, ipad
3	Facebook	Money, Marketing, Mobi, Free

## Pre-processing of textual data

Text-preprocessing is a significant technique for cleaning the raw data in a dataset, and it is the first and most important stage in removing extraneous text (Albalawi, Buckley & Nikolov, 2021; HaCohen-Kerner, Miller & Yigal, 2020). Before extracting features from text, it is necessary to eliminate any undesired data from the dataset. Unwanted data in the text dataset include punctuation, http links, special characters, and stop words.

**Figure 3:**
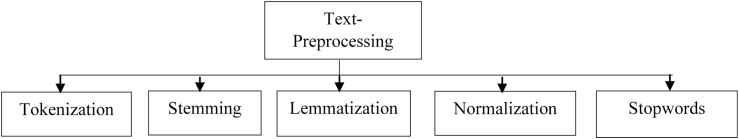
Various text-preprocessing techniques.

As illustrated in the Fig. 3, there are numerous text-preprocessing techniques available that can be used to remove superfluous information from incoming text input.

### Tokenization

It entails breaking down words into little components known as tokens. HTML tags, punctuation marks, and other undesirable symbols, for example, are removed from the text. The most widely used tokenization method is whitespace tokenization. The entire text is broken down into words during this procedure by removing whitespaces. To split the text into tokens, a well-known Python module known as “regular expressions” can be used, and it is frequently used to do Natural Language Processing (NLP) tasks. The following Table 6 depicts an example of a statement and its tokens.

**Table 6:** Illustration of a sentence and its generated tokens.

Sentence	Tokens
“I went to the library to read books”	“I”, “went”,”to”,”the”,”library”,”to”,”read”,”books”

### Stemming

It is concerned with the process of reducing words to their fundamental meanings; for instance, the terms drunk, drink, and drank are reduced to their root, drink. Stemming can produce non-meaningful terms that aren’t in the dictionary, and it can be accomplished using the Natural Language Tool Kit library in conjunction with PorterStemmer. Overstemming occurs when a significantly more chunk of a word is cut off than is required, resulting in words being incorrectly reduced to the same root word. Due to understemming, some words may be mistakenly reduced to more than one root word.

### Lemmatization

It employs lexical and morphological analysis, as well as a proper lexicon or dictionary, to link a term to its origin. The underlying word is known as a ‘Lemma,’ and words such as plays, playing, and played are all distinct variants of the word ‘play.’ So ‘play’ is the root word or ‘Lemma’ of all these words. The WordNet Lemmatizer is a Python Natural Language Tool Kit (NLTK) module that searches the WordNet Database for Lemmas. While lemmatizing, you must describe the context in which you want to lemmatize.

### Normalization

It is the process of reducing the number of distinct tokens in a text by reducing a term to its simplest version. It aids in text cleaning by removing extraneous information. By using a text normalization strategy for Tweets, Satapathy et al. (2017) were able to improve sentiment categorization accuracy by 4%.

### Stopwords removal

They are a category of frequently used terms in a language that have little significance. By removing these terms, we will be able to focus more on the vital facts. Stop words like “a,” “the,” “an,” and “so” are frequently used, and by deleting them, we may drastically reduce the dataset size. They can be successfully erased with the NLTK python library. Table 7 outlines some of the existing works on text spam detection that use various pre-processing techniques.

**Table 7:** Existing research on spam text pre-processing.

S.No	Authors	Pre-Processing technique used	Dataset	Classifier	Result
1	Méndez et al. (2005)	Tokenization, Stemming and Stopwords removal	e-mail text corpora	Support Vector Machine (SVM)	Classification accuracy is improved with pre-processing
2	Ruskanda (2019)	Stemming, Lemmatization,Stopwords removal and noise removal	Ling-spam *corpus* dataset with a total of 962 spam and ham messages	Naïve Bayes (NB) and Support Vector Machine (SVM)	Pre-processing with NB gives better results than SVM
3	Klassen (2013)	Data Normalization and discretization methods	Twitter dataset	SVM, Neural Networks (NN) and Random Forests (RF)	Overall classification rate of 84.30% is obtained
4	Jain et al. (2018)	Tokenization and Segmentation	1.5 million posts from real time Facebook data	NB, SVM and RF classifiers	RF classifier outperformed the others with a F-measure of
5	Ahmad, Rafie & Ghorabie (2021)	Stemming and Stopwords removal	Honeypot dataset with 2 million spam and non-spam tweets	Multilayer Perceptron (MLP), NB and RF	SVM outperformed others with a precision of 0.98 and an accuracy of 0.96

The descriptions and web URLs for some of the libraries or packages available for pre-processing text data are provided in Table 8 below.

**Table 8:** Tools available for pre-processing of spam text.

Library/Package	Description	Link
TextBlob	TextBlob is a Python text processing package. It provides a straightforward API for typical NLP tasks such as part-of-speech tagging and sentiment analysis.	https://textblob.readthedocs.io/en/dev/
Spacy	Spacy is a Python Natural Language Processing (NLP) package with a number of built-in features	https://spacy.io/
NLTK	The Natural Language Toolkit, or NLTK for short, is a Python-based set of tools and programmes for performing natural language processing.	https://www.nltk.org/
RapidMiner	Accessing and analysing various types of data, both organised and unstructured, is simplified.	https://rapidminer.com/products/studio/feature-list/
Memory-Based Shallow Parser	Can determine the grammatical structure of a sentence by parsing a string of letters or words using python	https://pypi.org/project/MBSP-for-Python/

For text pre-processing, researchers in the field of NLP use several methods provided in the NLTK package. They are open source which are simple to implement and they can also be used to execute other NLP-related applications.

## Feature-extraction techniques

Because many machine learning algorithms rely on numerical data rather than text, it is required to convert the text input into numerical vectors. This method’s goal is to extract meaningful information from a text that describes essential aspects of it.

### Bag of words (BoW)

The bag of words strategy is the most common and straightforward of all feature extraction procedures; it generates a word presence feature set from all of an instance's words. Each document is viewed as a collection or bag that contains all of the words. We may obtain a vector form that tells us the frequency of each word in a document, as well as repeated words in our document. Barushka & Hajek (2019) developed a spam review detection model that uses n-grams and the skip-gram word embedding method. They employed deep learning models to detect spam in 400 positive and negative hotel reviews from the TripAdvisor website. Table 8 (Term-document matrix) depicts the link between a document and its terms. The frequency of occurrence of a term in a group of documents is represented by each value in the Table 9.

**Table 9:** A bag of words illustration (BoW).

Words	Doc-1	Doc-2	Doc-3	Doc-4
Sentiment	2		3	2
Processing		2	4	1
Classification	1		2	
Algorithm		1	3	4

### N-grams

N-grams, which are continuous sequences of words or tokens in a document, are used in many Natural Language Processing (NLP) activities. They are classified into several types based on the values of ‘n,’ including Unigram (*n* = 1), Bigram (*n* = 2), and Trigram (*n* = 3). Kanaris, Kanaris & Stamatatos (2006) extracted n-gram characteristics from text using a dataset of 2,893 e-mails. They employed performance factors such as spam recall and precision in their study. They were able to construct a spam filtering approach with a precision score of more than 0.90 for spam identification by combining Support Vector Machine (SVM) with n-grams. They were able to construct a spam filtering approach with a precision score of more than 0.90 for spam identification by combining Support Vector Machine (SVM) with n-grams. Çıltık & Güngör (2008) proposed an efficient e-mail spam filtering technique to reduce time complexity, and they discovered that utilizing *n* = 50 for first n-words heuristics yielded improved results. The words in Table 10 below are instances of N-grams.

**Table 10:** An N-grams illustration.

S. No	Type of N-Gram	Example
1	Unigram	“I”, “Like”, “to”, “Play”, “Cricket”
2	Bi-gram	I Like, Like to, Play Cricket
3	Tri-gram	I Like to, to Play Cricket

### Term frequency-inverse document frequency (TF-IDF)

When employing bag of words, the terms with the highest frequency become dominant in the data. Domain-specific terms with lower scores may be eliminated or ignored as a result of this issue. This technique is performed by multiplying the number of times a word appears in a document (Term-Frequency-TF) by the term’s inverse document frequency (Inverse-Document Frequency-IDF) across a collection of documents. These scores can be used to highlight unique terms in a document or words that indicate crucial information. The computed TF-IDF score can then be fed into machine learning algorithms such as Support Vector Machines, which substantially improve the results of simpler methods such as Bag-of-Words. The values of TF and IDF is calculated as per the following Eqs. (1) and (2)

(1) $${\rm Tf}\left( {\rm w} \right) = {\rm \; }\displaystyle{{{\rm number\; of\; times\; \; in\; a\; document\; the\; word\; }\left( {\rm w} \right){\rm \; appears\; }} \over {{\rm total\; count\; of\; words\; in\; a\; document}}}$$

(2) $${\rm Idf}\left( {\rm w} \right) = {\rm Log}\displaystyle{{{\rm Total\; count\; of\; documents}} \over {{\rm Number\; of\; documents\; that\; contain\; the\; word\; w}}}\; \;$$

The Fattahi & Mejri (2020) examined the Bag of Words (BoW) and TF-IDF spam detection algorithms using text data containing 747 spam message instances. They used a variety of machine learning approaches to classify spam and were able to achieve an accuracy of 97.99% and precision of 98.97%. For spam text identification, they found just a minor difference in performance between the BoW and TF-IDF approaches.

### One hot encoding

Every word or phrase in the given text data is stored as a vector with only the values 1 and 0. Every word is represented by a separate hot vector, with no two vectors being identical. The sentence’s list of words can be defined as a matrix and implemented using the NLTK python package because each word is represented as a vector.

### Word embedding

One-hot encoding is ideal when we just have a little amount of data. Because the complexity develops substantially, we can use this method to encode a vast vocabulary. Comparable words have similar vector representations in word embedding, which is a form of word representation technique. Because each word is mapped to a different vector and the technique resembles a neural network, it is usually referred to as deep learning.

### Word2Vec

To process text made up of words, this approach transforms words into vectors and works in the same way as a two-layer network. Each word in the *corpus* is allocated a matching vector in the space. Word2vec employs either a continuous skipgram or a continuous bag of words architecture (CBOW). In the continuous skipgram, the current word is utilized to predict the neighboring words, whereas in the CBOW model, a middle word is predicted based on the surrounding or neighbouring words. The skip-gram model can accurately represent even rare words or phrases with a small quantity of training data, but the CBOW model is several times faster to train and has slightly better accuracy for common keywords. The word2vec approach has the advantage of allowing high-quality word embedding to be learned in less time and space. It makes it possible to learn larger embeddings (with greater dimensions) from a much larger *corpus* of text.

### Glove word embedding

It’s an unsupervised model for generating a vector for word/text representation. The distance between the terms is determined by their semantic similarity. Pennington, Socher & Manning (2014) were the first to use it to their studies. It employs a co-occurrence matrix, which shows how frequently words appear in a *corpus*, and is based on matrix factorization techniques. The Eq. (3) shows the calculation for the co-occurrence probability of the texts in each word embedding

(3) $$F\left( {{t_{a}},\ {t_{b}},\ {t_{c\; }}} \right) = \displaystyle{{{P_{ac}}} \over {{P_{bc}}}}$$

where,

The co-occurrence probability for the texts ${t_{a\; }}$ and ${t_{c\; }}$ is ${P_{ac}}$

The co-occurrence probability for the texts ${t_{b\; }}$ and ${t_{c\; }}$ is ${P_{bc}}$

The normal texts/words that appear in a document are ${t_{a\; }}$ and ${t_{b\; }}$ and the probe text is ${t_{c\; }}$

When the aforementioned ratio is ‘1’, the probe text is related to ${t_{a\; }}$ rather than ${t_{b\; }}$

Table 11 summarizes some of the existing research studies that use various feature extraction approaches such as TF-IDF, Bag of Words (BOW), N-grams, and Word embedding techniques such as Glove and Word2Vec.

**Table 11:** Existing works that employ various text feature extraction techniques.

S.No	Author	Dataset	Classification approach	Merits	Limitations	Result
1	Inuwa-Dutse, Liptrott & Korkontzelos (2018)	Honeypot, SPD manually and automatically annotated spam dataset	Support Vector Machine (SVM), Random Forest (RF), Multi-Layer Perception (MLP), Gradient Boosting and Max.Entropy	Real time spam detection is possible and the proposed feature set increases the system accuracy	Need to deal with the presence of lengthy tweets on spamming activity.	Accuracy-97.71% Precision-99% Recall-97% F-Score-98%
2	Aiyar & Shetty (2018)	13,000 comments from YouTube channels	RF, SVM, Naive Bayes (NB) with N-grams based features	Machine Learning (ML) models with N-grams has helped to improve the classification accuracy	The use of better word representation like Word2Vec is needed to improve system performance	F1-Score-0.97
3	Chu, Widjaja & Wang (2012)	774 spam campaigns in 1, 31,000 Tweets	RF, Decision Trees (DT), Decision Table, Random Tree, KStar, Bayes Net and Simple Logistic	Content and Behaviour features were combined to build an automatic spam detection model.	Need to explore more features to build a robust model for spam classification	Accuracy-94.5% FPR-4.1% FNR-6.6%
4	Alharthi, Alhothali & Moria (2021)	More than 10,000 Arabic tweets collected with Twitter API	Long Short Term Memory (LSTM) with word embedding feature representation	Time requirement to classify the tweets is very less compared to the state-of-the art methods	System classification accuracy depends on tweet length	Accuracy-0.97 Precision-0.98 Recall-0.95 F1-score-0.97
5	Liu, Pang & Wang (2019)	97,839 Restaurant (RES) and 31,317 Hotel review dataset (HOS)	Machine Learning (ML) techniques and Bi-LSTM	Could capture sophisticated spammer activities using multimodal neural network model	There is a need to analyze the use of other effective features to improve the performance	Recall-0.80 Precision-0.82 F1-score-0.81
6	Fusilier et al. (2015)	Hotel review *corpus* consisting of 1, 600 reviews	SVM, K-Nearest Neighbor and Naïve Bayes (NB)	Lexical content and stylistic information were captured better using character n-grams	Need to build a hybrid feature set combining character and word n-grams	F1-score-0.87
7	Wu et al. (2017)	10 day real-life Twitter dataset of 1,376,206 spam and 6,73,836 non-spam tweets	RF, Multi-Layer Perceptron (MLP) and Naïve Bayes	Variations in spamming activities are captured within a short span of time.	The model needs to be adaptable to new characteristics	Accuracy-99.35 Recall-91.03% Precision-95.84% F-measure-93.37%

## Spam text classification techniques

**Figure 4:**
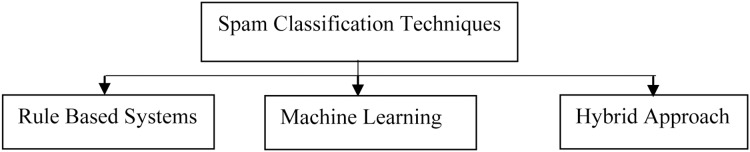
Various text-preprocessing techniques.

Text classifiers can organize and categorize practically any sort of material, including documents and internet text. Text classification is an important stage in natural language processing, with applications ranging from sentiment analysis to subject labelling and spam detection. Text classification can be done manually or automatically, however in the manual approach, a human annotator assesses the text’s content and categorizes it correctly. Machine learning techniques and other Artificial Intelligence (AI) technologies are used to automatically classify text in a faster and more accurate manner utilizing automatic text classification models. As shown in the Fig. 4 below, there are three techniques of classifying the text.

### Spam classification using rule based systems

They work by sorting the text into distinct groups using handcrafted linguistic rules. The entering text is classified using semantic factors based on its content. Certain terms can help you evaluate whether or not a text message is spam. The spam text has a few distinctive phrases that help differentiate it from non-spam language. The document is classified as spam when the number of spam words in it exceeds the number of non-spam (ham) terms. They operate by employing a set of framed rules, each of which is given a weight. The spam text *corpus* is scanned for spam content, and if any rules are found in the text, their weight is added to the overall score. Table 12 summarizes some of the existing works on spam classification using rule-based systems.

**Table 12:** Existing research works on spam classification using rule-based systems.

S.No	Author	Dataset	Classification approach	Merits	Limitations	Result
1	Shrivastava & Bindu (2014)	*Corpus* of 2,248 emails with 1,346 spam and ham texts	Rule based spam detection filter with some assigned weights	Combination of Genetic Algorithm with e-mail filtering methods facilitates efficient spam detection	Need to increase the size of dataset and in-depth analysis of parameters of Genetic algorithm is required	Accuracy-82.7% Precision-83.5%
2	Vanetti et al. (2013)	1,260 Facebook messages from Italian groups	Flexible rule-based system is used to customize the filtering criteria.	Automatic filtering of unwanted messages from Online Social Networks is made possible.	Care should be taken to handle the extraction of contextual features for better discrimination of samples.	Precision-81% Recall-93% F1-Score-87%
3	Saidani, Adi & Allili (2020)	Enron *Corpus* consisting of 2,893 messages with 2,412 ham and 481 ham text.	Manually and Automatically extracted rules from labelled emails	Domain categorization used in this work has helped to improve the filter performance	Continuous enhancement and updation of semantic features is needed.	Accuracy-0.98 Precision-0.98 Recall-0.98 F1-measure-0.97
4	Luo et al. (2011)	SpamAssassin *corpus* with 4,150 spam and 1,897 ham emails	Rule extraction, optimization and rule filtering models are used	Dynamic adjustment of static rules for improving the spam filter is made possible.	Value of threshold has an impact on classification performance and it has to be taken care of.	Accuracy-98.5% False Positive Rate-0.42% False Negative Rate-4.7%
5	Fuad, Deb & Hossain (2004)	Email *corpus* with 271 training and 30 test email text	Fuzzy Inference System with a set of Fuzzy rules	The system is made adaptive by making use of effective fuzzy rules.	Need to train the system with a large *corpus* to improve the accuracy.	Accuracy-90% Precision-83% Recall-72%

Based on the previous works on spam classification using rule-based techniques given in Table 12, we can conclude that rule-based techniques are well-appreciated by researchers for their importance in spam text classification. SpamAssassin is open source software that aids in the creation of rules for various categories and is preferred by spam detection researchers. Some rule-based systems rely on static rules that can’t be changed, so they can’t deal with constantly changing spam content. To improve the method’s ability to detect spam, the established rules must be updated on a regular basis. To deal with the varying nature of spam, the automatic rule generation concept can be used. For complex systems, rule-based systems have significant drawbacks in terms of time consumption, analysis complexity, and rule structuring. They also require more contextual features for effective spam detection, as well as a large training *corpus*.

### Machine Learning (ML) techniques for spam classification

To detect spam reviews, a variety of machine learning techniques have been deployed. There are two types of machine learning: supervised learning and unsupervised learning, both of which are extensively utilized in NLP applications. Jancy Sickory Daisy & Rijuvana Begum (2021) used the Nave Bayes method and the Markov Random Field to circumvent the limitations of other filtering algorithms. By combining two algorithms, this hybrid system was able to detect spam effectively while saving time and improving accuracy. Dedeturk & Akay (2020) compared the performance of their proposed spam filtering strategy, which is based on a logistic regression model, to that of existing models such as Support Vector Machine (SVM) and Naive Bayes (NB). They tested their algorithm on three publicly available e-mail spam datasets and discovered that it outperformed the others in spam filtering. Nayak, Amirali Jiwani & Rajitha (2021) employed a hybrid strategy that combined Nave Bayes and Decision Tree algorithms to identify spam e-mails (DT). They were able to obtain an accuracy of 88.12% using their hybrid approach. Table 12 covers a number of existing spam classification works that employ various Machine Learning (ML) methodologies. To protect social media accounts from spam, Sharma et al. (2021) used Decision Tree (DT) and K-Nearest Neighbor (K-NN) classifiers. They tested their method using the UCI machine learning e-mail spam dataset. With a classification accuracy of 90% and an F1-score of 91.5%, the Decision Tree classifier produced better results. In their research, Raza, Jayasinghe & Muslam (2021) found that multi-algorithm systems outperform single-algorithm systems when it comes to spam classification. For e-mail spam detection, they compared the performance of supervised and unsupervised machine learning algorithms. For better spam detection, the supervised approach outperformed the unsupervised approach. Junnarkar et al. (2021) used a two-step methodology to ensure that the mail people received was not spam. They utilized URL analysis and filtering to see if any of the links in the email were malicious or not. A total of five machine learning algorithms were investigated. On the e-mail spam dataset, Naive Bayes and Support Vector Machine achieved the highest accuracy of over 90%. The importance of machine learning techniques for spam text classification is studied by Al-Zoubi et al. (2018), Singh et al. (2021), Tang, Qian & You (2020) in their work in which they conclude that Machine Learning techniques overcome the drawbacks of rule-based techniques for spam content detection.

Based on the prior work on spam classification with Machine Learning approaches presented in Table 13, we can conclude that Machine Learning techniques are highly valued by researchers for their importance in spam text classification. Machine learning has the ability to adapt to changing conditions, and it can help overcome the limitations of rule-based spam filtering techniques. Support Vector Machines (SVM), a supervised learning model that analyses data and identifies patterns for classification, is among the most significant machine learning techniques. SVMs are straightforward to train, and some researchers assert that they outperform many popular social media spam classification methods. However, due to the computational complexities of the data input, the resilience and usefulness of SVM for high dimension data shrinks over time. Another machine learning algorithm that has been successfully used to detect spam in social media text is the decision tree. When it comes to training datasets, decision trees (DT) require very little effort from users. They suffer from certain disadvantages, such as the complexity of controlling tree growth without proper pruning and their sensitivity to over fitting of training data. As a consequence, they are rather poor classifiers and their classification accuracy is restricted. A Naive Bayes (NB) classifier simply applies Bayes’ theorem to the perspective classification of each textual data, assuming that the words in the text are unrelated to one another. Because of its simplicity and ease of use, it is ideal for spam classification and it could be used to detect spam messages in a variety of datasets with various features and attributes. An ensemble strategy, which combines various machine learning classifiers, can also be utilized to improve spam categorization jobs. We can deduce from various studies on Machine Learning for spam classification that ML techniques occasionally suffer from computational complexity and domain dependence. The researchers recommend Deep Learning (DL) techniques to avoid such limitations in ML techniques for spam classification because some algorithms take much longer to train and use large resources based on dataset.

**Table 13:** Existing research works on spam classification using machine learning.

S.No	Author	Dataset	Classification approach	Merits	Limitations	Result
1	Kontsewaya, Antonov & Artamonov (2021)	4,360 non-spam and 1,368 spam samples from the Kaggle Dataset	Logistic Regression (LR), Naïve Bayes (NB), K-Nearest Neighbor (K-NN) and Decision Trees(DT)	Presented a comparative analysis of different ML algorithms	Better DL based feature learning strategies can be employed for extracting relevant features.	Accuracy-0.99 Precision-0.97 Recall-0.99 F-measure-0.98
2	Mohammed et al. (2013)	Email-1,431 dataset	SVM, K-NN, NB and DT	Instead of using spam trigger words, which may fail, a lexicon-based approach is used to filter the data.	Less number of training samples used (272 ham and 1,219 spam). Need for a better feature extraction technique	Accuracy-85.96% Precision-84.5% F1-score-85.12
3	Watcharenwong & Saikaew (2017)	1,200 Labelled posts crawled from Facebook using a webcrawler	Random Forest (RF)	Social features like comments etc., are combined with textual features yields better results	Need to use image features to get improved results	Precision-98.19% Recall-98.12% F1-score-98.15%
4	Dhawan & Simran (2018)	25,847 Twitter users with 500K tweets are collected using Twitter API and a Web crawler	DT, NN, SVM, NB	Graph and Content based features extracted from Twitter aids in improving model’s performance	Need to analyze the use of Deep Learning (DL) techniques and bring in more metrics for performance evaluation.	Precision-1 Recall-0.41 F-measure-0.58
5	Ban et al. (2018)	Textual data collected from Twitter and Facebook with spam and on-spam content	SVM & NN	Hybrid architecture of SVM with NN helped to improve the classification results	Only a few performance metrics is evaluated to determine the model’s efficiency	Precision-85% Recall-84%
6	Dewan & Kumaraguru (2015)	4.4 million Facebook posts acquired using Graph API	RF	Automatic identification of spam text is done with 42 features using ML techniques	The labelled spam dataset was gathered through crowdsourcing and may be biased.	Accuracy-86.9% Precision-95.2%
7	Kumar et al. (2018)	Restaurant reviews from Yelp.com	LR, K-NN, NB, RF, SVM	For effective spam identification, uses both univariate and multivariate distribution across user ratings.	It is necessary to adjust the model to new characteristics and improve its efficiency.	Accuracy-0.76 F1-Score-0.79
8	Saeed, Rady & Gharib (2019)	Opinion spam *corpus* (DOSC & HARD) datasets with 1,600 opinion reviews in English	Rule-based and Machine learning classifiers (NB, SVM, K-NN, RF and NN)	The model’s performance was increased by using N-gram feature extraction and Negation handling.	Spam detection efficiency could be improved using Deep Learning (DL) techniques	Accuracy-95.25% Recall-91.75% Precision-98.66% F1-Score-95.08%
9	Mani et al. (2018)	Opinion spam *corpus* dataset with 1,600 reviews	NB, RF and SVM	The ensemble strategy aided in obtaining a higher accuracy score.	It is necessary to develop a control mechanism to reduce the propagation of fraudulent reviews.	Accuracy-87.68% Precision-0.89 Recall-0.85
10	McCord & Chuah (2011)	Random collection of tweets from 1,000 Twitter accounts containing both spam and non-spam text	RF, NB and K-NN	User and Content based features with RF classifier was successful in identifying spam and non-spam tweets	Need a larger Twitter dataset for evaluating the effectiveness of the model	Precision-95.97 Recall-0.95 F-measure-0.95

### Hybrid approach for spam classification

To increase spam classification performance, hybrid spam detection systems combine a machine learning-based classifier with a rule-based approach. To detect spam in emails, Abiramasundari (2021) utilized a hybrid technique that comprised “Rule Based Subject Analysis” (RBSA) and machine learning algorithms. Their rule-based solution involves assigning suitable weights to spam material and generating a matrix that is then submitted to a classifier. They tested their method on the Enron dataset (email *corpus*), and their proposed work with the SVM classifier achieved a very low positive rate of 0.03 with a 99% accuracy. Venkatraman, Surendiran & Arun Raj Kumar (2020) employed a semantic similarity technique combined with the Naive Bayes (NB) machine learning algorithm to classify spam material. The proposed “Conceptual Similarity Approach” computes the relationship between concepts based on their co-occurrence in the *corpus*. They tested their hybrid spam classification strategy using the Spambase and Enron *corpus* datasets. They have a near-perfect 98% accuracy rate. Wu (2009) used a novel technique to spam detection in their work, merging Neural Networks (NN) with rule-based algorithms. They classified spam content using Neural Networks, rule-based pre-processing, and behavior identification modules with an encoding approach. They tested their approach on an email *corpus* containing lakhs of emails and scored a 99.60% spam detection accuracy score.

## Deep learning (dl) approaches for spam classification

Deep learning models are gaining popularity among NLP researchers due to their ability to solve challenging problems (Kłosowski, 2018; Torfi et al., 2020). Deep learning is based on the idea of building a very large neural network inspired by brain activities and training it using a massive amount of data. They can cope with the scalability issue and extract the features from the data automatically. The most popular deep learning models among NLP researchers are Convolutional Neural Networks (CNN) and Long Short Tern Memory (LSTM) networks. Convolutional Neural Networks (CNN), one of the most important and extensively used Deep Learning approaches, has received a lot of attention in recent times for performing NLP tasks. It has been used successfully for sentiment analysis (Kim & Jeong, 2019), image (Sharma, Jain & Mishra, 2018) and text categorization (Song, Geng & Li, 2019), pattern recognition (Mo et al., 2019), and other tasks. For text categorization, Lai et al. (2015) used a recurrent structure to capture contextual information from textual data. Their technique was able to capture semantic information from text and outperformed CNN in classifying text texts. Tai, Socher & Manning (2015) employed the Long Short Term Memory Network (LSTM) to capture sequential information in textual data, and they built a tree LSTM model that could perform well for NLP applications. Basyar, Adiwijaya & Murdiansyah (2020) built a Long Short Term Memory (LSTM) network and a Gated Recurrent Unit (GRU) model to detect spam in the Enron e-mail spam dataset, which contained 34,519 records. The LSTM model outperformed the GRU model in spam detection, achieving an accuracy of 98.39%. Alauthman (2020) employed the Gated Recurrent Unit-Recurrent Neural Network (GRU-RNN) to recognize Botnet spam E-mails. On the SPAMBASE dataset, which included 4,601 spam and 2,788 non-spam e-mails, they achieved an accuracy of 98.7%. They evaluated the performance of GRU with several machine learning algorithms, but the GRU-based strategy produced the best results for spam detection. Hossain, Uddin & Halder (2021) used feature selection techniques including Heatmap, Recursive Feature Elimination, and Chi-Square feature selection techniques, along with Deep Learning models such as RNN, to select the most effective features for spam e-mail detection. On spam text information obtained from the UCI machine learning repository, they achieved a 99% accuracy. Tong et al. (2021) used a deep learning model based on LSTM and BERT to overcome issues such as unfair representation, inadequate detection effect, and poor practicality in Chinese spam detection. They created this model to capture complex text features using a long-short attention mechanism. In their work to detect spam reviews related to hotels, Liu et al. (2022) used a combination of Convolution structure and Bi-LSTM to extract important and comprehensive semantics in a document. They could be able to outperform current methods in terms of classification performance by achieving an F1-Score of around 92.8. There are many other research works (Crawford & Khoshgoftaar, 2021; Bathla & Kumar, 2021) employing Deep Learning (DL) techniques for spam detection that could capture contextual information of text for spam identification.

Based on the prior work on spam classification with Deep Learning approaches presented in Table 14. These Deep Learning techniques definitely helps in improving the performance of the spam detection model and also helps in reducing the effects of over-fitting that is seen in Machine Learning models. Unlike ML techniques, deep learning methods do not necessitate a manual feature extraction process or a large amount of computational resources. It can adapt to a wide range of spam content found in social media text and will be very effective at extracting spam data from the text. Based on previous research, we can deduce that combining word-embedding techniques with Deep Learning methods improves spam classification performance. However, with less training data, it is more difficult to avoid over-fitting, and the presence of unlabeled text in the input *corpus* will lower performance. The deep learning method is used to classify text that saves a lot of manpower and resources while also improving text classification accuracy.

**Table 14:** Existing research works on spam classification using deep learning.

S.No	Author	Dataset	Classification approach	Merits	Limitations	Result
1	Alom, Carminati & Ferrari (2020)	1. Twitter social honeypot dataset 2. Twitter 1KS-10KN dataset	Convolutional Neural Network (CNN)	Combination of tweet text with meta data has helped to attain good performance for spam classification	Using only textual data i.e tweets the system could not perform well	Accuracy-99.32% Precision-99.47% Recall-99.9% F1-Score-99.68%
2	Feng et al. (2018)	Sina Weibo dataset with 12,500 malicious URLs and 12,500 normal URLs	Convolutional Neural Network (CNN) with Word2Vec	Detects the spam content by utilizing low computing resources	Complexity of the model	Accuracy-91.36% false Positive Rate-8.82% and False Negative Rate-8.54%
3	AbdulNabi & Yaseen (2021)	Open source SpamBase dataset with 5,569 emails and Kaggle spam filter dataset	Fine-tuned BERT(Bidirectional Encoder Representations from Transformers) with Word2Vec approach	Spam detection efficiency is improved with the help of BERT word embedding approach	Need to utilize a large input sequence for better training of model.	Accuracy-0.98 F1-Score-0.98
4	Seth & Biswas (2017)	Image-Dataset with 1,521 spam images and 1,500 ham images. Text-Enron spam dataset	CNN with multimodal data (Image and Text)	Multimodal (Image+Text) technique helped to achieve greater accuracy compared to unimodal inputs	Need to improve the neural network model for achieving better accuracy by tuning the hyper parameters	Accuracy-98.11% F1-Score-0.98
5	Xu, Zhou & Liu (2021)	MicroblogPCU dataset-2,000 spam and non-spam data Weibo dataset-95,385 weibo tweets	Self-attention BiLSTM with ALBERT model-word vector model of BERT	Semantic and Contextual data from Tweets are captured using the Bi-LSTM model with self-attention mechanism	Computational time and resources required by the model has to be reduced.	Accuracy-0.91 Recall-0.89 F1-score-0.90
6	Ma et al. (In press)	Twitter and SinaWeibo datasets with 2,313 and 2,351 rumors	Recurrent Neural Networks (RNN) with extra hidden layers	RNN model with multiple hidden and embedding layers help to reduce the spam detection time.	Massive unlabeled data from social media reduces the system performance. Works well for Weibo dataset compared to Twitter	Accuracy-0.88 Precision-0.85 Recall-0.95 F1-Score-0.89
7	Neisari, Rueda & Saad (2021)	Single domain hotel review dataset with 800 reviews (Dataset1) Multi-domain dataset with 2,840 reviews (Dataset2)	Un-supervised Self Organized Maps (SOM) with CNN	Semantic information is captured well with the help of SOM to enhance the spam detection performance	Need to improve the performance of SOM model by including additional layers and features.	Accuracy-0.87 F1-measure-0.88
8	Shahariar et al. (2019)	Single domain hotel review dataset with 800 reviews and Yelp spam review dataset with 2,000 reviews	CNN and Bi-LSTM with Word2Vec method	Word2Vec approach has helped to get better feature vector representations to get efficient results.	Data labelling process need to be improved and requires more training samples (1,600 reviews) to improve the classification performance.	Accuracy-94.56% F1-measure-95.2%
9	Makkar & Kumar (2020)	WEBSPAM-2007 dataset containing 222 spam and 3,776 non-spam web pages.	LSTM model	It provides cognitive ability to search engine for automatic webspam detection.	Need to tune the algorithm to handle large scale data from web	Accuracy-96.96% F1-measure-94.89%
10	Zhuang et al. (2021)	WEBSPAM-UK2006 and WEBSPAM-UK2007 datasets with spam and non-spam labels	Deep Belief Networks (DBN)-Stacked Restricted Boltzmann Machine (RBM)	Algorithm’s performance is improved by employing a preference function which is based on DBN	Proposed algorithm’s performance is dependent on selection of appropriate reference examples.	Accuracy-0.94 Precision-0.95 Recall-0.95

## Challenges in spam detection/classification from social media content

Spam content on social media continues to rise as people’s use of social media grows dramatically. The technology underlying spam spread is amazing, and some social media sites were unable to correctly identify spam contents/spammers. Some legitimate social media users manufacture duplicates in order to communicate with a group of recognized pals. It is tough to distinguish between a spammer and a legitimate user with a duplicate profile. Spammers also employ many fake identities to distribute dangerous and fraudulent material, making it harder to track them down. A spammer may also employ social bots to automatically post messages based on the user’s interests. Many businesses use “crowdsourcing” to enhance production, in which some people are paid to offer false reviews about a product that is not good. The machine learning method for spam detection suffers from over-fitting and sometimes suffers from a lack of training samples. They may also encounter difficulties if the spammer is intelligent and quick enough to adapt. When the input dataset is quite large, ML approaches suffer from temporal complexity, and memory requirements are also an issue. If there are undesirable features in the dataset, the classifier’s performance suffers, and an efficient feature selection algorithm is required.

Unsupervised learning suffers from a storage shortage, as well as a scarcity of efficient spam detection methods. As a result, there is a strong need to pursue a method that is flexible and efficient, such as Deep Learning, in order to tackle the challenges encountered by traditional Machine Learning methodologies. Spammers also employ Deep Learning algorithms to manipulate social media material in order to generate spam. These bogus contents developed using Deep Learning algorithms are difficult to detect, necessitating more effort to resist them. If there is a shortage of properly annotated data available, the notion of transfer-learning might be used as an alternative to Machine Learning.

## Open issues and Future Directions

Some of the issues in spam detection are the presence of sarcastic text, multilingual data, and improper labelling of the datasets. Many researchers use APIs to gather data related to a given language and geographical area, there is a bias in the data collected through social media. Some studies employ raw data without much pre-processing, which results in duplicated features and lower classification performance. Some datasets exhibit a class imbalance, for example, the ‘spam’ class has a large number of samples whereas the ‘ham’ class has a small number of samples.

There are a limited number of labelled datasets available for spam text, as well as a limited number of attributes available in these text datasets, which is a problem. For efficient research, a dataset with correct labelling is required, as is large computational power in the case of a large dataset. Only a few studies have used deep learning techniques and semantic approaches to detect spam. Exploring the use of multimodal content (text and images) from social media for social media would be a significant future challenge.

## Conclusion

We have described numerous strategies for spam text identification in depth in our systematic literature review on spam content detection and categorization. Our research also looked into the various techniques for pre-processing, feature extraction, and spam text classification. This survey will assist researchers in conducting research in the field of social media spam detection as it highlights some of the best works done in this field. We’ve also provided details on a number of databases that can be used for spam detection studies. The various previous works on spam text pre-processing, feature extraction, and classification will aid researchers in determining the most appropriate strategies for their research in this area. In future development, we’d like to include some other spam detection approaches, as well as their benefits and drawbacks.

## Additional Information and Declarations

### Data Availability

The following information was supplied regarding data availability: This is a literature review.
